# Discovery of Potent Carbonic Anhydrase and Acetylcholinesterase Inhibitors: 2-Aminoindan β-Lactam Derivatives

**DOI:** 10.3390/ijms17101736

**Published:** 2016-10-20

**Authors:** Hayriye Genç, Ramazan Kalin, Zeynep Köksal, Nastaran Sadeghian, Umit M. Kocyigit, Mustafa Zengin, İlhami Gülçin, Hasan Özdemir

**Affiliations:** 1 Department of Chemistry, Faculty of Arts and Sciences, Sakarya University, Sakarya 54050, Turkey; hayriyegenc@sakarya.edu.tr (H.G.); mzengin@sakarya.edu.tr (M.Z.); 2 Department of Basic Science, Faculty of Science, Erzurum Technical University, Erzurum 25700, Turkey; rkalin@atauni.edu.tr; 3 Department of Chemistry, Faculty of Sciences, İstanbul Medeniyet University, İstanbul 34730, Turkey; zkoksal@atauni.edu.tr; 4 Department of Chemistry, Faculty of Science, Atatürk University, Erzurum 25240, Turkey; parham_taslimi_un@yahoo.com (N.S.); hozdemir@atauni.edu.tr (H.Ö.); 5 Vocational School of Health Services, Cumhuriyet University, Sivas 58140, Turkey; ukocyigit@cumhuriyet.edu.tr; 6 Department of Zoology, College of Science, King Saud University, Riyadh 11451, Saudi Arabia

**Keywords:** carbonic anhydrase, acetylcholinesterase, β-lactam, 2-azetidinone, enzyme inhibition, enzyme purification

## Abstract

β-Lactams are pharmacologically important compounds because of their various biological uses, including antibiotic and so on. β-Lactams were synthesized from benzylidene-inden derivatives and acetoxyacetyl chloride. The inhibitory effect of these compounds was examined for human carbonic anhydrase I and II (hCA I, and II) and acetylcholinesterase (AChE). The results reveal that β-lactams are inhibitors of hCA I, II and AChE. The *K_i_* values of β-lactams (**2a**–**k**) were 0.44–6.29 nM against hCA I, 0.93–8.34 nM against hCA II, and 0.25–1.13 nM against AChE. Our findings indicate that β-lactams (**2a**–**k**) inhibit both carbonic anhydrases (CA) isoenzymes and AChE at low nanomolar concentrations.

## 1. Introduction

The β-lactams can be classified into several groups according to their structural characteristics, but their unique structural feature is the presence of the four-membered β-lactam (2-azetidinone) ring [1]. 2-Azetidinone containing antibiotics are known as β-lactam antibiotics and they are the most widely employed family of antibacterial agents [2]. Moreover, they have been reported as having antibacterial, anticancer, and antiviral activity, and an enzyme inhibition effect [3,4,5]. The investigation of the chemistry and biology of β-lactam continue to appeal to synthetic and medicinal organic chemists [6]. They have also been used for the preparation of different heterocyclic compounds of biological significance [7].

Carbonic anhydrases (CAs, E.C. 4.2.1.1) catalyse a very simple but physiologically essential reaction in all life kingdoms: the hydration of carbon dioxide (CO_2_) and water (H_2_O) to bicarbonate (HCO_3_^−^) and protons (H^+^) with a high efficiency [8,9,10,11]. CAs are metalloenzymes that participate in the control of pH in the body, are encoded by six different independent gene families (α-, β-, γ-, δ -, ζ-, and η-CA) and are found in eukaryotic and prokaryotic cells [12,13,14,15]. They catalyse reversible CO_2_ hydration, which are essential important physiologic processes in all life kingdoms [16,17,18].

$${\mathrm{CO}}_{2}+{\;H}_{2}O\;\overset{\mathrm{CA}}{\Leftrightarrow }H_{2}{\mathrm{CO}}_{3}\Leftrightarrow {\mathrm{HCO}}_{3}^{-}+{\;H}^{+}$$

Living organisms possess sixteen CA isoenzymes, which differ in their subcellular localization and catalytic activity [19,20,21,22]. They were found in various organs and tissues with different kinetic and molecular properties, expression levels, and oligomeric rearrangements as well as various abilities to respond to different inhibitory classes [23,24,25]. These enzymes play a very important role in different tissues [26,27]. CA I, II, III, VII, and XIII isoenzymes are cytosolic, CA IV, IX, XII, and CA XIV are bound to membranes, CA VA-VB are mitochondrial, and CA VI are in the milk and saliva [28,29]. The erythrocytes contain CA I, and II at high concentrations. The CA inhibitors (CAIs) are used as a class of pharmaceuticals, which used as diuretics, anti-glaucoma agents. Also, they used for treatment of gastric and duodenal ulcers, neurological disorders, and osteoporosis [30,31,32]. Up until now, the inhibitory effects against different CAs types have been investigated for different sulphonamides derivatives, heavy metal ions, phenols, antibiotics, and various drugs [33,34,35]. β-Lactams are widely used in the treatment of many diseases. However, there is no detailed study on erythrocytes hCA I, and II isoenzymes of β-lactams (**2a**–**k**).

Acetylcholinesterase (AChE) is a crucial enzyme used for transmission control between neurons [36] when the process is either mediated or modulated by the neurotransmitter acetylcholine (ACh). ACh is released by the axon terminal or varicosities of the transmitter neuron into the extracellular space to interact with the receptors of the other neuron. AChE hydrolyses ACh to choline and acetate [37,38]. If AChE is inhibited in the central nervous system, then the concentration of ACh increases in the synaptic cleft, leading to cholinergic crisis, which affords several dangerous effects, such as convulsions and respiratory problems, which could lead to death. AChE inhibitors (AChEIs) have medical applications and are particularly important for the symptomatic treatment of Alzheimer’s disease (AD) to enhance central cholinergic transmission [39]. AD is a fatal and chronic neurodegenerative disease that usually starts slowly and gets worse over time [19]. From this perspective, there is a great need for improved AChEIs that show low toxicity, good brain penetration, and high bioavailability. The use of AChEIs to block the cholinergic degradation of acetylcholine (Ach) is therefore considered to be a promising approach for the treatment of AD [40,41].

In the present study, we investigated the inhibition profile of a series of β-lactam derivatives (**2a**–**k**) against CA I, and II isoforms from human erythrocytes and AChE enzyme.

## 2. Results and Discussion

**Figure 1:**
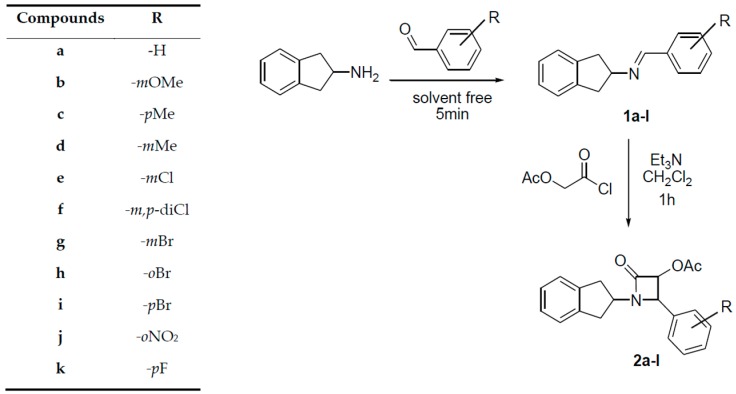
General synthesis route of novel β-lactam derivatives (**2a**–**k**).

β-Lactam derivatives are drugs that protect against many different gram positive-negative and anaerobic organisms. They are perhaps among the best-studied and most widely used antibiotics in the world [42]. The general synthesis route of the novel β-lactam derivatives (**2a**–**k**) is shown in Figure 1. 

**Figure 2:**
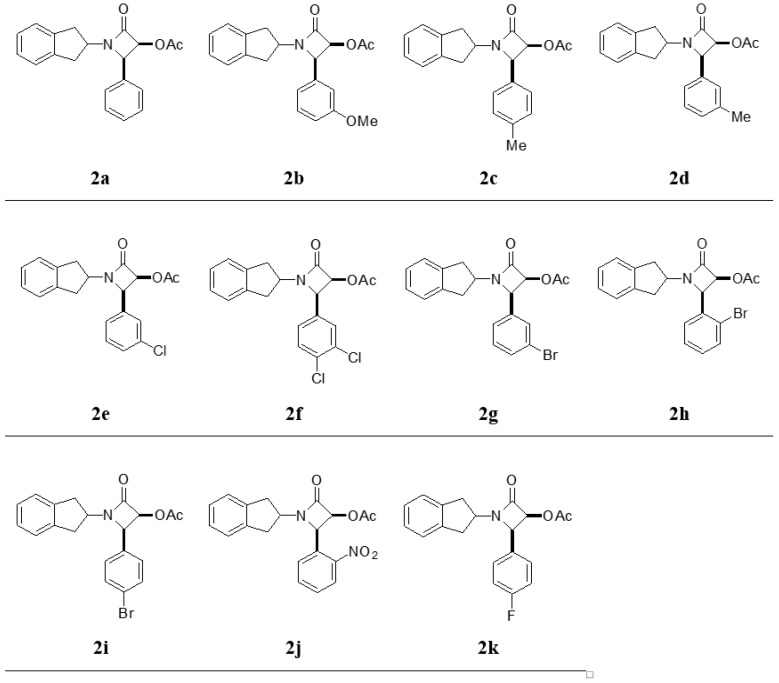
The chemical structures of the investigated β-lactam derivatives (**2a**–**k**).

Herein, β-lactam derivatives (**2a**–**k**) were prepared from benzylidene-inden derivatives and ketene and characterized by NMR and MS. We assumed that the synthesized compounds were in *cis* form based on the literature [43,44,45]. The chemical structures of **2a**–**k** are given in Figure 2. The in vitro inhibitory effects of compounds **2a**–**k** were also examined for purified hCA I, and II isoenzymes activities using the esterase activity method. β-Lactam derivatives inhibit growth of sensitive bacteria by inactivating enzymes in the cell membrane. β-Lactam derivatives were synthesized and evaluated as inhibitors of protease, elastase, and the cysteine protease papain. Some drug molecules are enzyme inhibitors, so their discovery is important in biochemistry research [46]. Inhibitors of CA have several medical applications, such as in treating glaucoma disease, high blood pressure, and the neurological disorders epilepsy and Alzheimer’s disease. Some research groups are currently working on the synthesis of new inhibitors of the carbonic anhydrase family for the treatment of some diseases [47,48,49].

**Figure 3:**
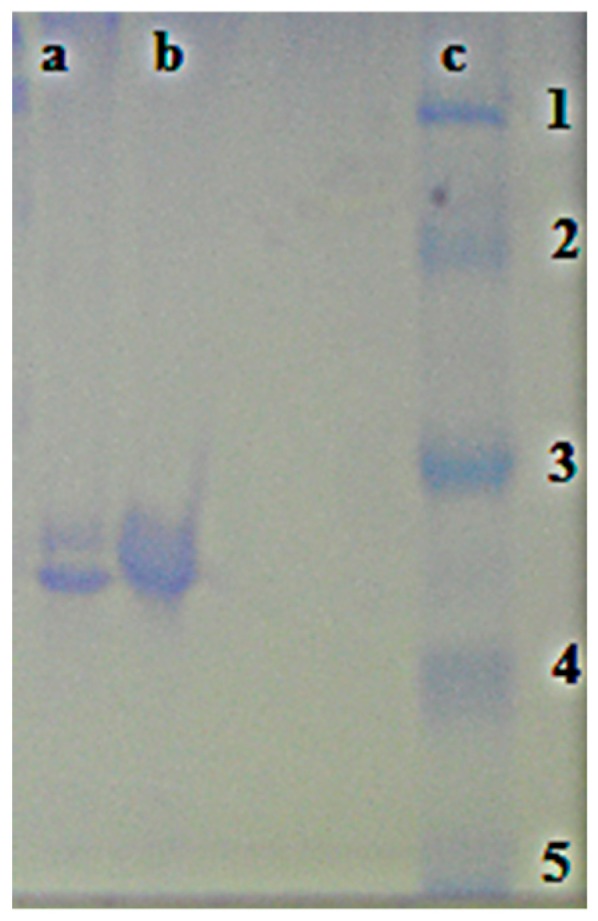
Sodium dodecyl sulphate-polyacrylamide gel electrophoresis bands of carbonic anhydrase I and II isoenzymes and standard proteins. Lanes **a**: hCA II, **b**: hCA I, **c**: standard proteins; standards **1**: 116 kDa (β-Galactosidase from *E. Coli*), **2**: 97 kDa (Phosphorilase from rabbit muscle), **3**: 66 kDa (Bovine serum albumin), **4**: 45 kDa (Ovalbumin), **5**: 29 kDa (carbonic anhydrase from bovine erythrocyte).

Some chemicals at low dosages are effective by altering normal enzyme activity and by inhibiting a specific enzyme [50,51]. It is well known that β-lactams had inhibition properties on hCA I, and II isoenzymes and are used in therapies [52,53]. The inhibition effects of newly synthesized compounds **2a**–**k** were determined for the first time against hCA I, and II. For this purpose, as shown in Table 1, hCA I and II were separately purified from erythrocytes with affinity chromatography. The hCA I was purified 127.9-fold with a specific activity of 1151.4 EU/mg and overall yield of 63.9%, and the hCA II enzyme was purified 788.9-fold with a specific activity of 7100.0 EU/mg and overall yield of 56.4% (Table 1). The purification was monitored by SDS-PAGE. After this process, a single band was observed for each isoenzyme (Figure 3). For the compounds, the inhibitor concentrations causing up to 50% inhibition (IC_50_ values) were determined from the regression analysis graphs. From in vitro studies, it is understood that hCA I, hCA II, and AChE were inhibited by these β-lactam compounds **2a**–**k** (Table 2). The inhibition data of β-lactam derivatives **2a**–**k** reported here are shown in Table 2, and the following comments can be drawn from these data:

(1) Cytosolic hCA I is expressed in the body and can be found in high concentrations in the blood and gastrointestinal tract. All β-lactam derivatives **2a**–**k** exhibited effective inhibitory activity against the cytosolic isoenzyme hCA I with a *K_i_* value of 0.35 ± 0.105–6.29 ± 2.068 nM (Table 1). Also, β-lactam derivative **2g** shown the most powerful CA I inhibition effect with a *K_i_* value of 0.35 ± 0.105 nM. On the other hand, we found that acetazolamide (AZA), which is used as a clinical CA inhibitor in the treatment of glaucoma, cystinuria, epilepsy, altitude sickness, periodic paralysis, dural ectasia, idiopathic intracranial hypertension, and central sleep apnoea [52], has a *K_i_* value of 170.34 ± 2.48 nM (Table 2). The results clearly show that all β-lactam derivatives **2a**–**k** demonstrate more effective hCA inhibitory activity than that of AZA.

(2) With regard to the profiling assay against cytosolic hCA II, β-lactam derivatives **2a**–**k** have similar inhibition effects; with *K_i_* values ranging from 0.93 ± 0.295 through 8.34 ± 3.530 nM. For comparison, AZA, which is used as a clinical CA inhibitor showed a *K_i_* value of 115.43 ± 1.63 nM. This result clearly shows that all β-lactam derivatives **2a**–**k** are a rather effective inhibitor for the cytosolic isoform hCA II. The most powerful CA II inhibition effect was found in β-lactam derivatives of **2i** with a *K_i_* value of 0.93 ± 0.295 nM. 

(3) The compounds or drugs possessing AChE inhibitory effects are used for the treatment of AD. However, these drugs and compounds have many undesired side effects. Also, the utilization and development of new effective AChEIs is highly desired. Currently the most prescribed AChEIs are Tacrine, Galantamine, Rivastigmine, and Donepezil [55]. In the present study, AChE was very effectively inhibited by β-lactam derivatives **1**–**11**, with *K_i_* value in the range of 0.25 ± 0.019–1.13 ± 0.472 nM (Table 2) and calculated from Lineweaver-Burk plots [56]. On the other hand, Tacrine had a *K_i_* value of 3.90 ± 0.792 nM.

## 3. Materials and Methods

### 3.1. Chemicals

CN-Br-activated Sepharose-4B, *p*-nitrophenylacetate, and chemicals for electrophoresis were purchased from Sigma-Aldrich Co. (Steinheim, Germany). All other chemicals were of analytical grade and obtained from Merck (Darmstadt, Germany).

### 3.2. General Procedure for the Synthesis of Imines

2-Amino indane (1 eq) and benzaldehyde (1 eq) were stirred in a beaker for five minutes. The resulting crude imine product was recrystallized from dichloromethane/hexane to give target compound in 95%–99% yield. General synthesis route of novel β-lactam derivatives (**2a**–**k**) is given in Figure 1.

*(E)-N-Benzylidene-2,3-dihydro-1H-inden-2-amine* (**1a**). Yield 98%; ^1^H NMR (300 MHz; ppm; CDCl_3_) δ 3.14 (dd, *J* = 16.05, 7.36 Hz, 2H), 3.21 (dd, *J* = 8.0, 16.3 Hz, 2H), 4.32 (p, *J* = 7.1 Hz, 1H), 7.16–7.26 (m, 4H), 7.39–7.43 (m, 3H), 7.74–7.77 (m, 2H), 8.39 (1H, s); ^13^C NMR (75 MHz; ppm; CDCl_3_) δ 41.2 (2C), 71.5, 124.7, 126.7 (2C), 128.4, 128.8, 130.83, 136.5, 142.2 (2C), 160.1.

*(E)-N-(3-Methoxybenzylidene)-2,3-dihydro-1H-inden-2-amine* (**1b**). Yield 96%; ^1^H NMR (300 MHz; ppm; CDCl_3_) δ 3.12 (dd, *J* = 15.8, 7.0 Hz, 2H), 3.20 (dd, *J* = 8.41, 16.4 Hz, 2H), 3.82 (3H, s), 4.26 (p, *J* = 7.14 Hz, 1H), 6.94 (dt, *J* = 7.62, 1.66 Hz, 1H), 7.14–7.35 (m, 7H), 8.33 (s, 1H); ^13^C NMR (75 MHz; ppm; CDCl_3_) δ 41.1 (2C), 55.6, 71.4, 111.9, 117.5, 121.7, 124.7 (2C), 126.7 (2C), 129.8 (2C), 137.9, 142.3 (2C), 160.1.

*(E)-N-(4-Methylbenzylidene)-2,3-dihydro-1H-inden-2-amine* (**1c**). Yield 97%; ^1^H NMR (300 MHz; ppm; CDCl_3_) δ 2.36 (3H, s), 3.13 (dd, *J* = 15.3, 6.9 Hz, 2H), 3.17 (dd, *J* = 7.3, 15.4 Hz, 2H), 4.27 (p, *J* = 7.2 Hz, 1H), 7.14–7.23 (m, 6H), 7.63 (d, *J* = 7.99 Hz, 2H), 8.32 (s, 1H); ^13^C NMR (75 MHz; ppm; CDCl_3_) δ 21.8, 41.2 (2C), 71.6, 124.7 (2C), 126.7 (2C), 128.4 (2C), 129.5 (2C), 133.8, 141.1, 142.3 (2C), 160.1.

*(E)-N-(3-Methylbenzylidene)-2,3-dihydro-1H-inden-2-amine* (**1d**). Yield 97%; ^1^H NMR (300 MHz; ppm; CDCl_3_) δ 3.10 (dd, *J* = 16.0, 7.4 Hz, 2H), 3.19 (dd, *J* = 7.6, 15.8 Hz, 2H), 3.81 (3H, s), 4.26 (p, *J* = 7.20 Hz, 1H), 7.26–7.42 (m, 6H), 7.61–7.63 (d, *J* = 6.27 Hz, 1H), 7.73 (s, 1H), 8.43 (1H, s); ^13^C NMR (75 MHz; ppm; CDCl_3_) δ 21.6, 41.3 (2C), 71.7, 124.8 (2C), 126.2, 126.8 (2C), 128.7, 128.8, 131.8, 136.5, 138.6, 142.3 (2C), 160.5.

*(E)-N-(3-Chlorobenzylidene)-2,3-dihydro-1H-inden-2-amine* (**1e**). Yield 98%; ^1^H NMR (300 MHz; ppm; CDCl_3_) δ 3.19 (dd, *J* = 15.7, 6.4 Hz, 2H), 3.26 (dd, *J* = 7.3, 15.7 Hz, 2H), 4.36 (p, *J* = 6.9 Hz, 1H), 7.32–7.44 (m, 6H), 7.63 (d, *J* = 6.64 Hz, 1H), 7.84 (1H, s), 8.35 (1H, s); ^13^C NMR (75 MHz; ppm; CDCl_3_) δ 41.2 (2C), 71.4, 124.8 (2C), 126.8 (2C), 126.8 (2C), 128.0, 130.1, 130.8, 135.0, 138.3, 142.1, 158.6.

*(E)-N-(3,4-Dichlorobenzylidene)-2,3-dihydro-1H-inden-2-amine* (**1f**). Yield 98%; ^1^H NMR (300 MHz; ppm; CDCl_3_) δ 3.11 (dd, *J* = 15.6, 6.7 Hz, 2H), 3.18 (dd, *J* = 7.3, 15.6 Hz, 2H), 4.28 (p, *J* = 7.0 Hz, 1H), 7.15–7.24 (m, 4H), 7.35 (dd, *J* = 8.50, 2.26 Hz, 2H), 7.66 (dd, *J* = 8.50, 2.26 Hz, 2H), 8.30 (1H, s); ^13^C NMR (75 MHz; ppm; CDCl_3_) δ 41.2 (2C), 71.5, 124.8 (2C), 126.8 (2C), 129.1 (2C), 129.7 (2C), 134.9, 136.7, 142.1 (2C), 158.8.

*(E)-N-(3-Bromobenzylidene)-2,3-dihydro-1H-inden-2-amine* (**1g**). Yield 95%; ^1^H NMR (300 MHz; ppm; CDCl_3_) δ 3.11 (dd, *J* = 15.6, 6.5 Hz, 2H), 3.19 (dd, *J* = 7.3, 15.6 Hz, 2H), 4.29 (p, *J* = 6.9 Hz, 1H), 7.15–7.27 (m, 5H), 7.51 (d, *J* = 7.67 Hz, 1H), 7,61 (d, *J* = 7.67 Hz, 1H), 7.93 (1H, s), 8.29 (1H, s); ^13^C NMR (75 MHz; ppm; CDCl_3_) δ 41.1 (2C), 71.4, 123.1, 124.7 (2C), 126.7 (2C), 127.3, 130.3, 130.9, 133.7, 138.5, 142.1 (2C), 158.5.

*(E)-N-(2-Bromobenzylidene)-2,3-dihydro-1H-inden-2-amine* (**1h**). Yield 97%; ^1^H NMR (300 MHz; ppm; CDCl_3_) δ 3.17 (dd, *J* = 15.6, 6.4 Hz, 2H), 3.26 (dd, *J* = 7.2, 15.7 Hz, 2H), 4.42 (p, *J* = 6.8 Hz, 1H), 7.13–7.36 (m, 6H), 7.59 (dd, *J* = 7.67, 1.69 Hz, 1H), 8.08 (dd, *J* = 7.66, 1.43 Hz, 1H), 8.77 (1H, s). ^13^C NMR (75 MHz; ppm; CDCl_3_) δ 41.2 (2C), 71.3, 124.7 (2C), 125.2, 126.7 (2C), 127.8, 129.2, 131.9, 133.2, 134.8, 142.1 (2C), 159.1.

*(E)-N-(4-Bromobenzylidene)-2,3-dihydro-1H-inden-2-amine* (**1i**). Yield 98%; ^1^H NMR (300 MHz; ppm; CDCl_3_) δ 3.11 (dd, *J* = 15.6, 6.6 Hz, 2H), 3.19 (dd, *J* = 7.3, 15.6 Hz, 2H), 4.29 (p, *J* = 6.9 Hz, 1H), 7.15–7.24 (m, 4H), 7.52 (dd, *J* = 8.4, 5.0 Hz, 2H), 7.59 (dd, *J* = 8.6, 4.8 Hz, 2H), 8.30 (1H, s). ^13^C NMR (75 MHz; ppm; CDCl_3_) δ 41.1 (2C), 71.4, 124.7 (2C), 125.1, 126.7 (2C), 129.8 (2C), 132.0 (2C), 135.3, 142.1 (2C), 158.9.

*(E)-N-(2-Nitrobenzylidene)-2,3-dihydro-1H-inden-2-amine* (**1j**). Yield 97%; ^1^H NMR (300 MHz; ppm; CDCl_3_) δ 3.13 (dd, *J* = 15.7, 5.9 Hz, 2H), 3.25 (dd, *J* = 7.2, 15.7 Hz, 2H), 4.39 (p, *J* = 6.6 Hz, 1H), 7.15–7.25 (m, 4H), 7.56 (dt, *J* = 7.46, 1.54 Hz, 2H), 8.02 (dd, *J* = 7.71, 1.39 Hz, 2H), 8.9 (1H, s); ^13^C NMR (75 MHz; ppm; CDCl_3_) δ 41.1 (2C), 71.2, 124.5, 124.8 (2C), 126.8 (2C), 130.1, 130.8, 131.5, 133.7, 141.9 (2C), 149.0, 156.2.

*(E)-N-(4-Fluorobenzylidene)-2,3-dihydro-1H-inden-2-amine* (**1k**). Yield 94%; ^1^H NMR (300 MHz; ppm; CDCl_3_) δ 3.11 (dd, *J* = 15.6, 6.7 Hz, 2H), 3.18 (dd, *J* = 7.3, 15.6 Hz, 2H), 4.28 (p, *J* = 7.0 Hz, 1H), 7.03–7.24 (m, 6H), 7.71 (dd, *J* = 4.9, 5.5 Hz, 2H), 7.59 (dd, *J* = 8.6, 4.9 Hz, 2H), 8.32 (1H, s); ^13^C NMR (75 MHz; ppm; CDCl_3_) δ 41.2 (2C), 71.3, 115.7 (2C), 116.0 (2C), 124.7 (2C), 126.7 (2C), 130.2, 130.3, 142.2 (2C), 158.6.

### 3.3. General Procedure for the Synthesis of β-Lactams

To a solution of imine (1 eq) and triethylamine (3 eq) in dichloromethane, a solution of acetoxyacetyl chloride (2 eq) in dichloromethane was added dropwise over a period of 10 min at room temperature. The reaction mixture was then stirred for an additional 1 h at room temperature. The mixture was concentrated, then extracted with Ethyl Acetate and dried over Magnesium Sulphate; the solvent was removed in a vacuum. Obtained product was purified over a silica gel packed column chromatography using Hexane: EtOAc (1:1 *v*/*v*). The purified product was dried under vacuo and recrystallized from Ethanol yields β-lactam derivatives (70%–93% yield).

### 3.4. Spectral Data

(3S*,4R*)-1-(2,3-Dihydro-1H-inden-2-yl)-2-oxo-4-phenylazetidin-3-yl acetate (**2a**). Yield 91%, m.p. 171–173 °C; ^1^H NMR (300 MHz; ppm; CDCl_3_) δ 1.65 (3H, s), 2.88 (dd, *J* = 15.8, 6.1 Hz, 1H), 2.89 (dd, *J* = 15.8, 7.1 Hz, 1H), 3.14–3.28 (m, 2H), 4.54 (p, *J* = 7.0 Hz, 1H), 4.81 (d, *J* = 4.6 Hz, 1H), 5.72 (d, *J* = 4.6 Hz, 1H), 6.93–6.96 (m, 1H), 7.04–7.21 (m, 5H), 7.29–7.31 (m, 3H); ^13^C NMR (75 MHz; ppm; CDCl_3_) δ 20.1, 37.3, 37.4, 54.0, 61.7, 76.6, 124.6 (2C), 127.0 (2C), 128.3 (2C), 128.5 (2C), 128.9, 133.7, 140.3, 140.3, 165.3, 169.2; MS: *m*/*z* 344.10 [M + Na]^+^.

(3S*,4R*)-1-(2,3-Dihydro-1H-inden-2-yl)-2-(3-methoxyphenyl)-4-oxoazetidin-3-yl acetate (**2b**). Yield 70%, m.p. 98–100 °C; ^1^H NMR (300 MHz; ppm; CDCl_3_) δ 1.70 (3H, s), 2.90 (dd, *J* = 15.9, 6.1 Hz, 1H), 2.99 (dd, *J* = 15.8, 7.1 Hz, 1H), 3.13–3.27 (m, 2H), 3.75 (s, 3H), 4.54 (p, *J* = 6.6 Hz, 1H), 4.77 (d, *J* = 4.7 Hz, 1H), 5.72 (d, *J* = 4.7 Hz, 1H), 6.66–6.67 (1H, m), 6.77–6.85 (2H, m), 6.96 (d, *J* = 6.94 Hz, 1H), 7.04–727 (m, 4H); ^13^C NMR (75 MHz; ppm; CDCl_3_) δ 20.2, 37.3, 37.4, 54.0, 55.4, 61.6, 76.5, 113.8, 114.6, 120.8, 124.5, 124.6 (2C), 127.0 (2C), 129.4, 135.4, 140.4, 159.5, 165.2, 169.3; MS: *m*/*z* 344.16 [M + Na]^+^.

(3S*,4R*)-1-(2,3-Dihydro-1H-inden-2-yl)-2-oxo-4-p-tolylazetidin-3-yl acetate (**2c**). Yield 92%, m.p. 113–115 °C; ^1^H NMR (300 MHz; ppm; CDCl_3_) δ 1.68 (3H, s), 2.29 (3H, s), 2.88 (dd, *J* = 15.8, 6.0 Hz, 1H), 2.98 (dd, *J* = 15.8, 7.1 Hz, 1H), 3.13–3.27 (m, 2H), 4.53 (p, *J* = 6.6 Hz, 1H), 4.76 (d, *J* = 4.6 Hz, 1H), 5.71 (d, *J* = 4.6 Hz, 1H), 6.92–7.27 (m, 8H); ^13^C NMR (75 MHz; ppm; CDCl_3_) δ 20.1, 21.5, 37.3, 37.4, 54.0, 61.7, 76.5, 124.5, 125.6, 127.0 (2C), 128.1, 129.3, 129.6, 133.6, 138.0, 140.4 (2C), 165.3, 169.3; MS: *m*/*z* 358.04 [M + Na]^+^.

(3S*,4R*)-1-(2,3-Dihydro-1H-inden-2-yl)-2-oxo-4-m-tolylazetidin-3-yl acetate (**2d**). Yield 87%, m.p. 96–98 °C; ^1^H NMR (300 MHz; ppm; CDCl_3_) δ 1.69 (3H, s), 2.34 (3H, s), 2.87 (dd, *J* = 15.8, 6.4 Hz, 1H), 2.97 (dd, *J* = 15.7, 7.3 Hz, 1H), 3.18 (m, *J* = 7.2, 15.3 Hz, 1H), 3.22 (m, *J* = 6.3, 15.3 Hz, 1H), 3.75 (3H, s), 4.51 (p, *J* = 6.8 Hz, 1H), 4.79 (d, *J* = 4.6 Hz, 1H), 6.95-6.97 (m, 1H), 7.02–7.17 (m, 7H); ^13^C NMR (75 MHz; ppm; CDCl_3_) δ 20.2, 21.4, 37.2, 37.3, 53.9, 61.6, 76.6, 124.6 (2C), 127.0, 127.0, 128.5 (2C), 129.0 (2C), 130.6, 138.7, 140.4, 140.4, 165.4, 169.4; MS: *m*/*z* 358.09 [M + Na]^+^.

(3S*,4R*)-2-(3-Chlorophenyl)-1-(2,3-dihydro-1H-inden-2-yl)-4-oxoazetidin-3-yl acetate (**2e**). Yield 98%, m.p. 115–117 °C; ^1^H NMR (300 MHz; ppm; CDCl_3_) δ 1.78 (3H, s), 2.83 (dd, *J* = 15.9, 7.0 Hz, 1H), 3.00 (dd, *J* = 15.9, 5.2 Hz, 1H), 3.10–3.24 (m, 2H), 4.57 (p, *J* = 6.1 Hz, 1H), 4.72 (d, *J* = 4.6 Hz, 1H), 5.71 (d, *J* = 4.6 Hz, 1H), 6.89–6.91 (m, 1H), 7.01–7.28 (m, 7H); ^13^C NMR (75 MHz; ppm; CDCl_3_) δ 20.1, 37.5, 37.6, 54.0, 61.1, 76.6, 124.5, 124.6, 126.7, 127.1, 127.2, 128.6, 129.1, 129.5, 134.3, 136.0, 140.2, 164.9, 169.2; MS: *m*/*z* 358.20 [M + 2H]^+^.

(3S*,4R*)-2-(3,4-Dichlorophenyl)-1-(2,3-dihydro-1H-inden-2-yl)-4-oxoazetidin-3-yl acetate (**2f**). Yield 84%, m.p. 138–140 °C; ^1^H NMR (300 MHz; ppm; CDCl_3_) δ 1.77 (3H, s), 2.79 (dd, *J* = 4.4, 16.0 Hz, 1H), 3.01 (dd, *J* = 16.0, 3.8 Hz, 1H), 3.10 (dd, *J* = 6.6, 17.1 Hz, 1H), 3.18 (dd, *J* = 4.6, 16.0 Hz, 1H), 4.56 (p, *J* = 6.2 Hz, 1H), 4.76 (d, *J* = 4.6 Hz, 1H), 5.70 (d, *J* = 4.6 Hz, 1H), 6.87–6.90 (m, 1H), 6.97 (d, *J* = 8.28 Hz, 1H), 7.04–7.14 (m, 4H), 7.33 (d, *J* = 8.28 Hz, 1H); ^13^C NMR (75 MHz; ppm; CDCl_3_) δ 20.3, 37.5, 37.9, 54.0, 60.5, 76.5, 124.5, 124.6, 127.2, 127.3, 127.7, 130.2, 130.5, 132.6, 133.0, 134.3, 140.1, 140.1, 164.8, 169.2; MS: *m*/*z* 392.20 [M + H]^+^.

(3S*,4R*)-2-(3-Bromophenyl)-1-(2,3-dihydro-1H-inden-2-yl)-4-oxoazetidin-3-yl acetate (**2g**). Yield 78%, m.p. 132–134 °C; ^1^H NMR (300 MHz; ppm; CDCl_3_) δ 1.73 (3H, s), 2.83 (dd, *J* = 4.9, 16.0 Hz, 1H), 3.00 (dd, *J* = 15.9, 7.0 Hz, 1H), 3.17 (ddd, *J* = 5.2, 6.5, 15.6 Hz, 2H), 4.58 (p, *J* = 6.0 Hz, 1H), 4.70 (d, *J* = 4.6 Hz, 1H), 5.70 (d, *J* = 4.6 Hz, 1H), 6.89–6.92 (m, 1H), 7.05–7.17 (m, 4H), 7.26–7.27 (m, 2H), 7.40–7.43 (m, 1H); ^13^C NMR (75 MHz; ppm; CDCl_3_) δ 20.1, 37.5, 37.6, 54.0, 61.0, 76.5, 122.3, 124.5, 124.5, 127.1, 127.3, 129.8, 131.5, 132.0, 136.3, 140.2, 140.2, 164.9, 169.2; MS: *m*/*z* 424.05 [M + H + Na]^+^.

(3S*,4R*)-2-(2-Bromophenyl)-1-(2,3-dihydro-1H-inden-2-yl)-4-oxoazetidin-3-yl acetate (**2h**). Yield 83%, m.p. 123–125 °C; ^1^H NMR (300 MHz; ppm; CDCl_3_) δ 1.69 (3H, s), 2.92 (dd, *J* = 6.0, 15.8 Hz, 1H), 3.02 (dd, *J* = 15.8, 7.0 Hz, 1H), 3.22 (d, *J* = 6.2 Hz, 2H), 4.53 (p, *J* = 6.5 Hz, 1H), 5.40 (d, *J* = 4.7 Hz, 1H), 5.85 (d, *J* = 4.7 Hz, 1H), 6.99–7.01 (m, 1H), 7.06–7.19 (m, 4H), 7.29–7.38 (m, 2H), 7.47–7.50 (m, 1H); ^13^C NMR (75 MHz; ppm; CDCl_3_) δ 20.1, 37.2, 37.5, 54.2, 60.6, 76.0, 124.5, 124.6, 124.7, 126.9, 127.1, 127.2, 129.4, 130.1, 133.0, 133.3, 140.1, 140.2, 165.5, 168.9; MS: *m*/*z* 422.03 [M + Na]^+^.

(3S*,4R*)-2-(4-Bromophenyl)-1-(2,3-dihydro-1H-inden-2-yl)-4-oxoazetidin-3-yl acetate (**2i**). Yield 76%, m.p. 114–116 °C; ^1^H NMR (300 MHz; ppm; CDCl_3_) δ 1.73 (3H, s), 2.83 (dd, *J* = 5.3, 15.9 Hz, 1H), 2.99 (dd, *J* = 15.9, 7.0 Hz, 1H), 3.15 (m, *J* = 5.7, 13.2 Hz, 1H), 3.18 (m, *J* = 6.6, 13.2 Hz, 1H), 4.56 (p, *J* = 6.2 Hz, 1H), 4.74 (d, *J* = 4.6 Hz, 1H), 6.92 (m, 1H), 7.02 (d, *J* = 8.28 Hz, 2H), 7.06–7.16 (3H, m), 7.41 (d, *J* = 8.41 Hz, 2H); ^13^C NMR (75 MHz; ppm; CDCl_3_) δ 20.2, 37.5, 37.5, 54.0, 61.1, 76.5, 122.9, 124.5, 124.6, 127.1 (2C), 130.1 (2C), 131.5 (2C), 133.0, 140.2, 140.2, 165.0, 169.2; MS: *m*/*z* 421.92 [M + Na]^+^.

(3S*,4R*)-1-(2,3-Dihydro-1H-inden-2-yl)-2-(2-nitrophenyl)-4-oxoazetidin-3-yl acetate (**2j**). Yield 93%, m.p. 117–119 °C; ^1^H NMR (300 MHz; ppm; CDCl_3_) δ 1.70 (3H, s), 2.99 (dd, *J* = 5.6, 15.9 Hz, 1H), 3.08 (dd, *J* = 15.8, 6.9 Hz, 1H), 3.25 (d, *J* = 6.1 Hz, 2H), 4.57 (p, *J* = 6.6 Hz, 1H), 5.59 (d, *J* = 5.2 Hz, 1H), 6.04 (d, *J* = 5.2 Hz, 1H), 6.97–6.99 (m, 1H), 7.04–7.12 (m, 3H), 7.46–7.52 (m, 1H), 7.59–7.63 (m, 2H), 7.95–7.98 (m, 1H); ^13^C NMR (75 MHz; ppm; CDCl_3_) δ 20.1, 37.2, 37.7, 54.7, 57.4, 76.4, 124.4, 124.6, 125.3, 127.2, 127.4, 129.4, 129.5, 130.3, 132.9, 139.9, 140.1, 149.0, 165.9, 168.6; MS: *m*/*z* 388,93 [M + Na]^+^.

(3S*,4R*)-1-(2,3-Dihydro-1H-inden-2-yl)-2-(4-fluorophenyl)-4-oxoazetidin-3-yl acetate (**2k**). Yield 91%, m.p. 114–116 °C; ^1^H NMR (300 MHz; ppm; CDCl_3_) δ 1.71 (3H, s), 2.84 (dd, *J* = 5.5, 15.8 Hz, 1H), 2.99 (dd, *J* = 15.9, 7.1 Hz, 1H), 3.18 (d, *J* = 6.2 Hz, 2H), 4.56 (p, *J* = 6.3 Hz, 1H), 4.77 (d, *J* = 4.6 Hz, 1H), 5.70 (d, *J* = 4.6 Hz, 1H), 6.91–6.99 (m, 3H), 7.04–7.16 (m, 5H); ^13^C NMR (75 MHz; ppm; CDCl_3_) δ 20.1, 37.4, 37.5, 53.9, 61.0, 76.6, 115.2, 115.5, 124.5, 124.5, 127.1 (2C), 129.6, 129.6, 130.2, 130.3, 140.2 (2C), 165.1, 169.2; MS: *m*/*z* 344.13 [M + Na]^+^.

### 3.5. Biochemical Assays

Erythrocytes were obtained from the Research Hospital at Atatürk University. The red cells were haemolysed with ice-cold water after washing with 0.9% NaCl. The hemolysate was applied to the prepared Sepharose-4B-tyrosine-sulfanylamide affinity gel [57]. Both CA isoenzymes were purified by Sepharose-4B-l-tyrosine-sulfanilamide affinity chromatography in a single step [58,59,60]. Sepharose-4B-l-tyrosine-sulfanilamide was prepared according to a previous method. Thus, homogenate solution acidity was adjusted and supernatant was transferred to the previously prepared Sepharose-4B-l-tyrosine-sulphanilamide affinity column [61,62]. The proteins flow in the column eluates was spectrophotometrically determined at 280 nm. All purification steps were performed at 4 °C. Protein quantity was determined at 595 nm according to the Bradford method [63]. Bovine serum albumin was used as the standard protein [64,65,66]. To monitor purification of both isoenzymes, sodium dodecyl sulphate-polyacrylamide gel electrophoresis (SDS-PAGE) was used according to the procedure of Laemmli [67]. In this application, the imaging method was performed in 10% and 3% acrylamide for the running and the stacking gel, respectively, with 0.1% SDS [68,69,70]. 

Enzyme activity was determined hydrolysis of *p*-nitrophenylacetate (PNA) to *p*-nitrophenolate at 348 nm according to the method of Verpoorte et al. [71] and described previously. The inhibitory effects of β-lactam derivatives **2a**–**k** were examined. To obtain the half maximal inhibitory concentration (IC_50_) values, CA I, and II activity was measured in the presence of β-lactam derivatives at different cuvette concentrations. Activity (%)-[β-lactam] graph was drawn for each of the β-lactam derivatives **2a**–**k** [72,73,74]. To determine *Ki* values, three different β-lactam derivative concentrations were tested. In these experiments, different substrate (PNA) concentrations were used and Lineweaver-Burk curves were drawn [56] as previously described [54].

The inhibition effects of β-lactam derivatives **2a**–**k** on AChE activities were measured according to the Ellman’s method [75] described previously [76]. Acetylthiocholine iodide (AChI) and 5,5′-dithio-bis(2-nitro-benzoic) acid (DTNB) were used as substrate. To this end, 100 μL of Tris/HCl buffer (1 M, pH 8.0) and 10 μL of β-lactam derivative solution at different concentrations and 50 μL AChE (5.32 × 10^−3^ U) solution were mixed and incubated for 10 min at 25 °C. Then 50 μL of DTNB (0.5 mM) was transferred. Then the reaction was initiated by the addition of 50 μL of AChI. The hydrolysis of AChI was recorded spectrophotometrically by the formation of 5-thio-2-nitrobenzoate anion as the result of the reaction of DTNB with thiocholine at a wavelength of 412 nm [77]. The IC_50_ values were determined by spectrophotometric measurement of the effect of increasing test compound (β-lactam derivatives **2a**–**k**) concentrations on AChE activity. The IC_50_ and *K_i_* values are calculated in the same way as for CA isoenzymes. Tacrine was used as positive control.

## 4. Conclusions

The hCA I, and II isoenzymes were inhibited by β-lactams **2a**–**k** at different functional groups (CH_3_, NO_2_, Br, F, Cl, and phenol) in the micromolar range. These compounds have shown nanomolar inhibition against both cytosolic hCA I, and II. These results indicate that the β-lactam ring and derivatives may be new CA inhibitors in addition to the well-known sulphonamides. Also, AChE was potently inhibited by β-lactams **2a**–**k** with *K_i_* values in the range of 0.25 ± 0.019–1.13 ± 0.472 nM.

## Figures and Tables

**Table 1:** Summary of purification procedure for human carbonic anhydrase (hCA) I, and II from fresh human erythrocytes with Sepharose-4B-l-tyrosine-sulphanilamide affinity chromatography.

Purification Steps		Volume (mL)	Total Enzyme Activity (EU)	Total Protein (mg)	Specific Activity (EU/mg)	Yield (%)	Purification Fold
Hemolysate		50	6300	700	9.0	100	1
Sepharose-4B-l-tyrosine-sulphanilamide affinity chromatography	hCA I	10	4030	3.5	1151.4	63.9	127.9
	hCA II	5	3550	0.50	7100.0	56.4	788.9

**Table 2:** Human carbonic anhydrase isoenzymes (hCA I and II) inhibition value with β-lactam derivatives **2a**–**k** by an esterase assay with *p*-nitrophenylacetate (NPA) as a substrate.

Compounds	IC_50_ (nM)						*K_i_* (nM)		
	hCA I	*R^2^*	hCA II	*R^2^*	AChE	*R^2^*	hCA I	hCA II	AChE
**2a**	0.612	0.9578	5.212	0.9099	0.885	0.9960	0.44 ± 0.115	3.54 ± 0.405	1.13 ± 0.472
**2b**	2.303	0.9906	6.418	0.9009	0.913	0.9868	1.41 ± 0.547	3.15 ± 1.139	0.39 ± 0.069
**2c**	2.107	0.9400	3.707	0.9012	0.783	0.9913	1.50 ± 0.657	2.96 ± 0.157	0.55 ± 0.136
**2d**	0.231	0.9704	4.176	0.9029	0.634	0.9926	1.49 ± 0.290	3.26 ± 0.708	0.77 ± 0.041
**2e**	3.984	0.9107	5.825	0.9136	0.668	0.9724	6.29 ± 2.068	8.34 ± 3.530	0.42 ± 0.020
**2f**	1.627	0.9936	5.975	0.9230	0.602	0.9791	1.16 ± 0.514	1.50 ± 0.421	0.46 ± 0.045
**2g**	0.652	0.9000	3.809	0.9295	0.734	0.9803	0.35 ± 0.105	3.39 ± 1.158	0.44 ± 0.057
**2h**	2.646	0.9264	5.212	0.9662	0.450	0.9947	0.91 ± 0.143	2.19 ± 0.921	0.25 ± 0.019
**2ı**	2.548	0.9359	4.030	0.9604	0.705	0.9984	0.97 ± 0.245	0.93 ± 0.295	0.36 ± 0.045
**2j**	4.814	0.9284	5.023	0.9057	0.704	0.9746	1.09 ± 0.136	2.88 ± 1.168	0.56 ± 0.073
**2k**	2.502	0.9886	6.863	0.9132	0.859	0.9939	0.94 ± 0.430	1.34 ± 0.539	0.68 ± 0.117
AZA ^Ψ,^*	101.19	0.9509	113.75	0.9791	-	-	170.34 ± 2.48	115.43 ± 1.63	-
**TAC ^⌘^**	-	-	-	-	4.101	0.9951	-	-	3.90 ± 0.792

**^Ψ^** Acetazolamide (AZA) was used as a standard inhibitor for both carbonic anhydrase (CA) isoenzymes; **^⌘^** Tacrine (TAC) was used as a standard inhibitor for all AChE; * These values were obtained from [54].

## Supplementary Materials

Supplementary materials can be found at www.mdpi.com/1422-0067/17/10/1736/s1.
