# Supplementary material for: Discovery of Potent Carbonic Anhydrase and Acetylcholinesterase Inhibitors: 2-Aminoindan β-Lactam Derivatives
Source: Int J Mol Sci. 2016 Oct 20;17(10):1736. doi: 10.3390/ijms17101736 (PMC5085765; doi:10.3390/ijms17101736)
Supplement: Supplementary file 1 [file ijms-17-01736-s001.pdf]

Supplementary Material: Discovery of Potent Carbonic Anhydrase and Acetylcholinesterase Inhibitors: 2-Aminoindan β-Lactam Derivatives

Hayriye Genç, Ramazan Kalin, Zeynep Köksal, Nastaran Sadeghian, Umit M. Kocyigit, Mustafa Zengin, İlhami Gülçin and Hasan Özdemir

**Comp.**	**Lineweaver-Burk Graph**		
	**hCA I**	**hCA II**	**AChE**
**2a**	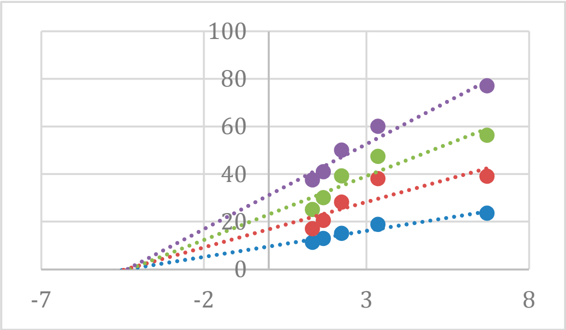	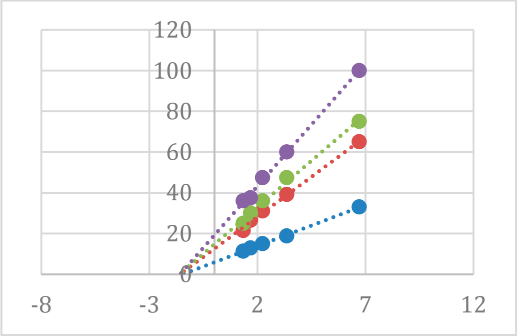	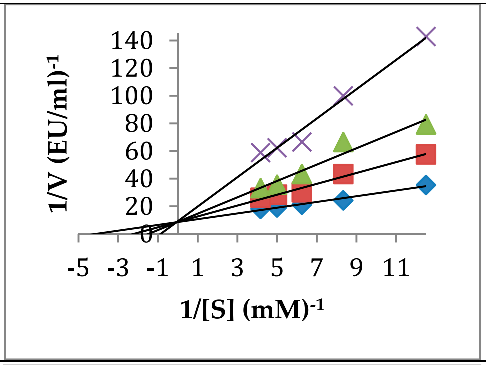
**2b**	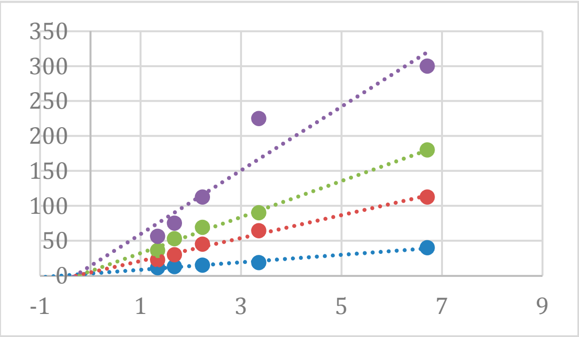	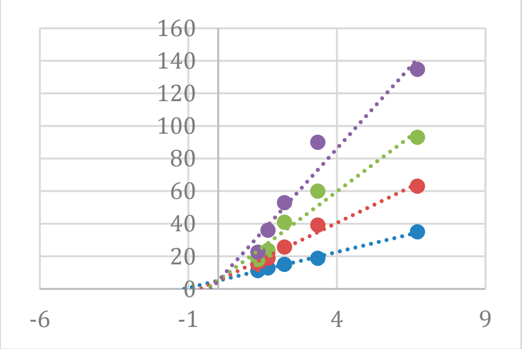	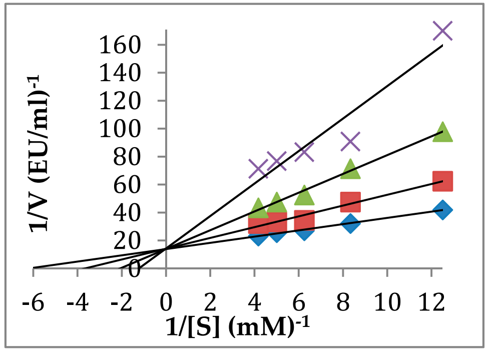

2c

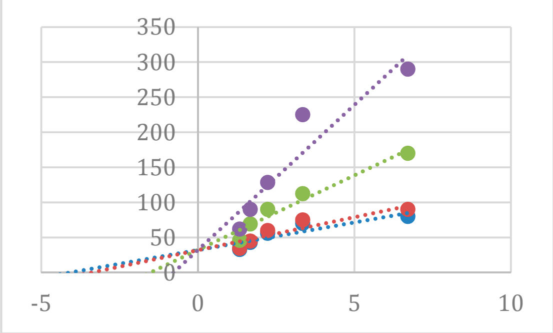
350
300
250
200
150
100
50
0
-5
0
5
10

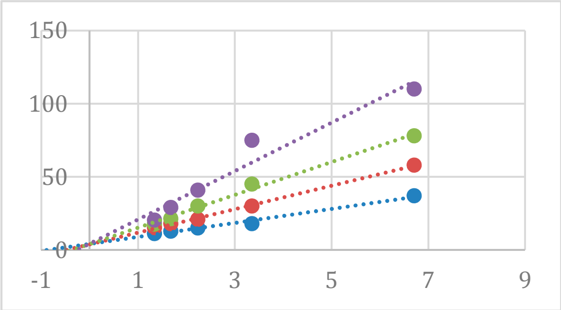
150
100
50
0
-1
1
3
5
7
9

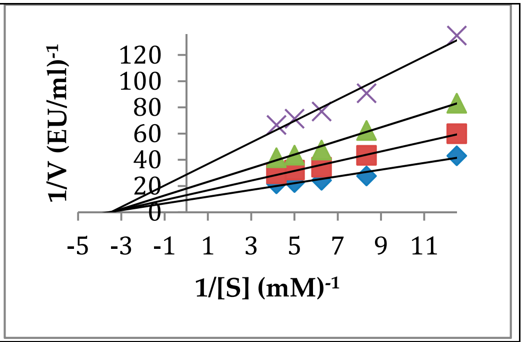
1/V (EU/ml)-1
120
100
80
60
40
20
0
-5
-3
-1
1
3
5
7
9
11
1/[S] (mM)-1

2d

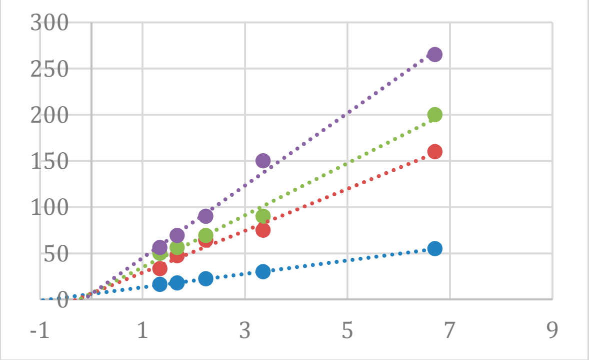
300
250
200
150
100
50
0
-1
1
3
5
7
9

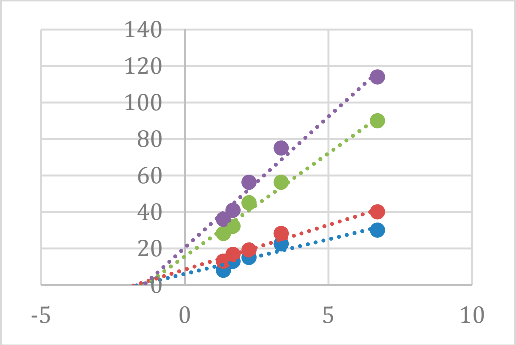
140
120
100
80
60
40
20
0
-5
0
5
10

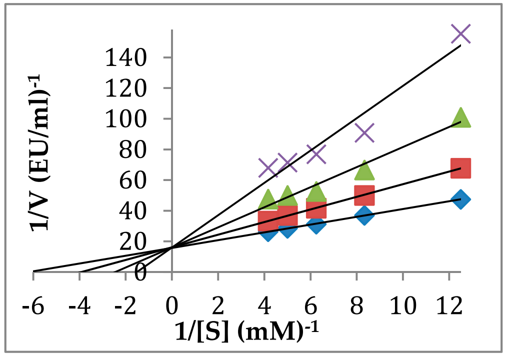
1/V (EU/ml)-1
140
120
100
80
60
40
20
0
-6
-4
-2
0
2
4
6
8
10
12
1/[S] (mM)-1

2e

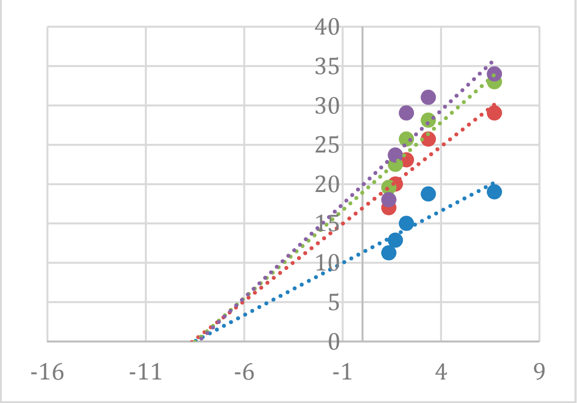
40
35
30
25
20
15
10
5
0
-16
-11
-6
-1
4
9

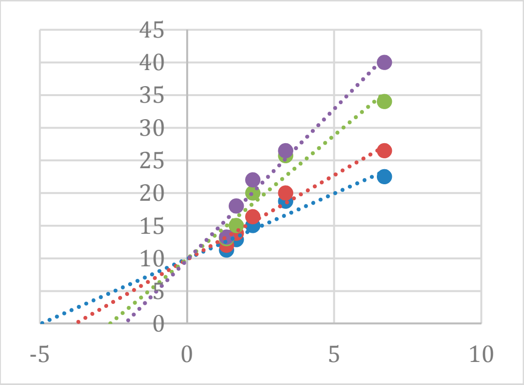
45
40
35
30
25
20
15
10
5
0
-5
0
5
10

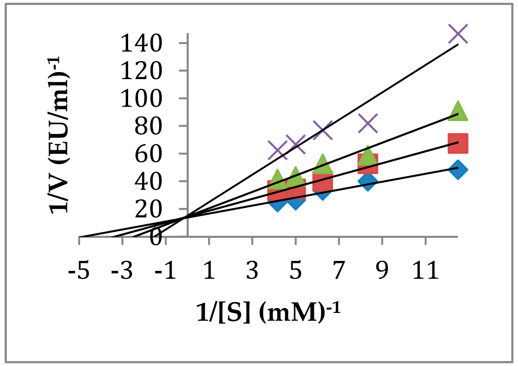
1/V (EU/ml)-1
140
120
100
80
60
40
20
0
-5
-3
-1
1
3
5
7
9
11
1/[S] (mM)-1

<table>
<tr><td>2f</td><td>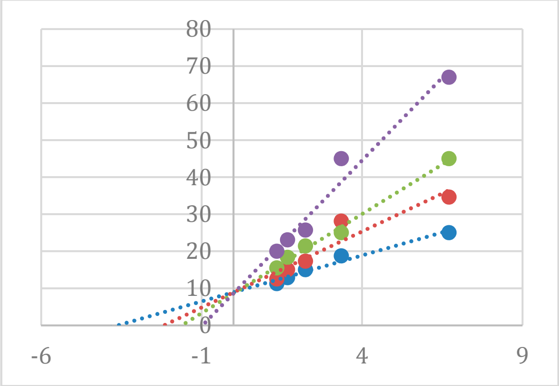</td><td>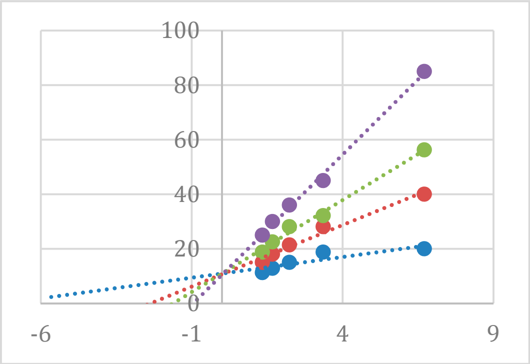</td><td>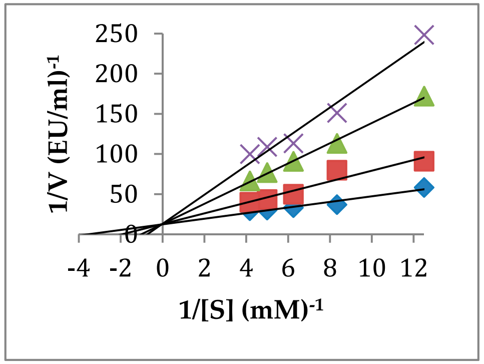
</td></tr>
<tr><td>2g</td><td>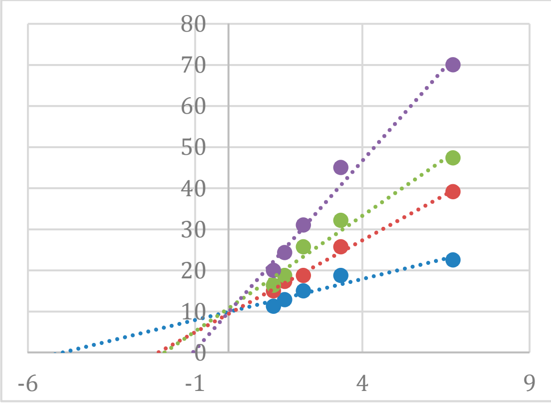</td><td>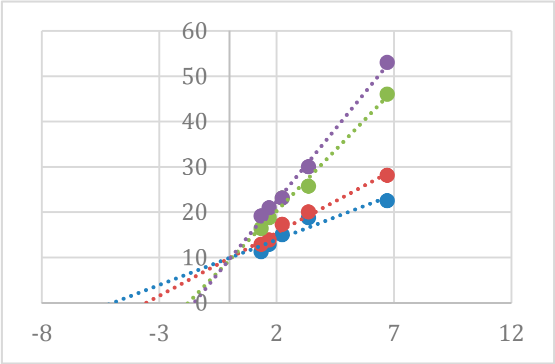</td><td>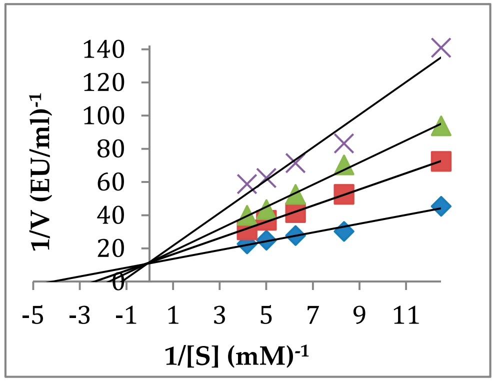
</td></tr>
<tr><td>2h</td><td>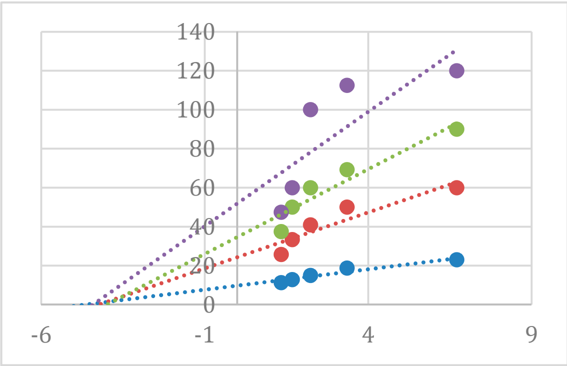</td><td>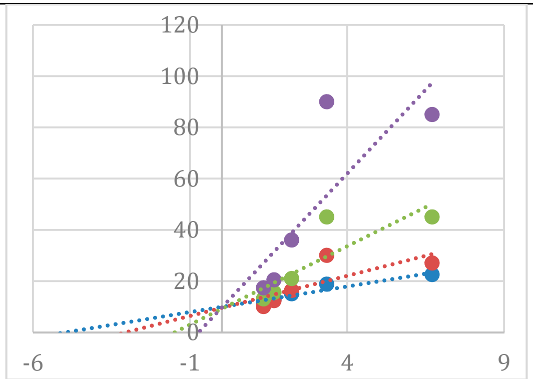</td><td>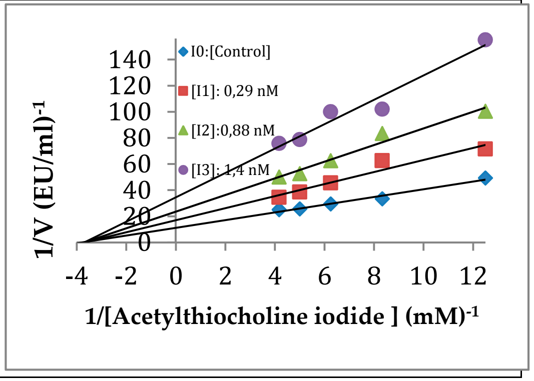
</td></tr>
</table>

2l

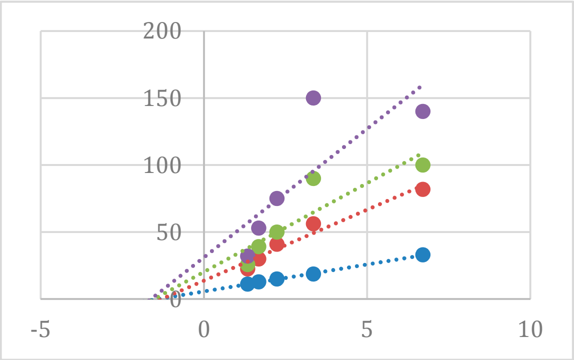
200
150
100
50
0
-5
0
5
10

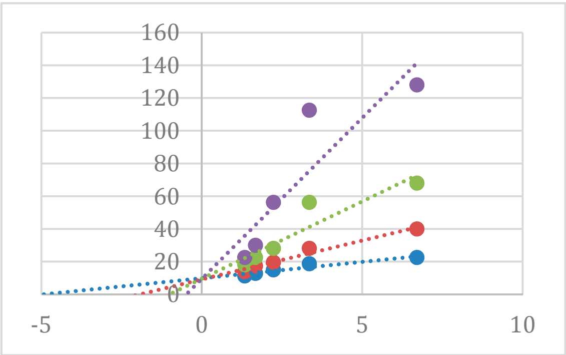
160
140
120
100
80
60
40
20
0
-5
0
5
10

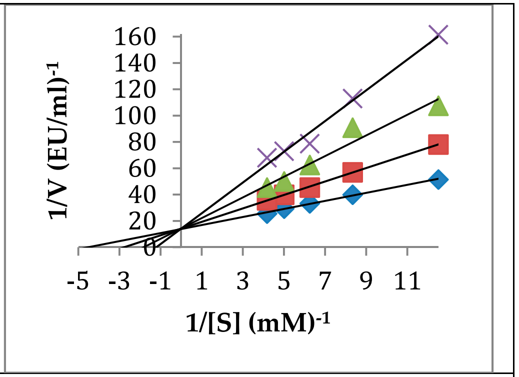
160
140
120
100
80
60
40
20
0
-5 -3 -1 1 3 5 7 9 11
1/V (EU/ml)-1
1/[S] (mM)-1

2j

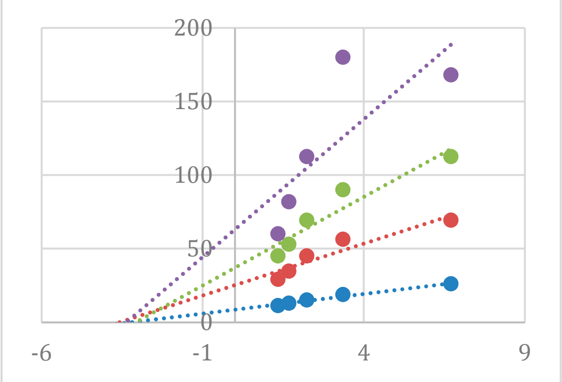
200
150
100
50
0
-6
-1
4
9

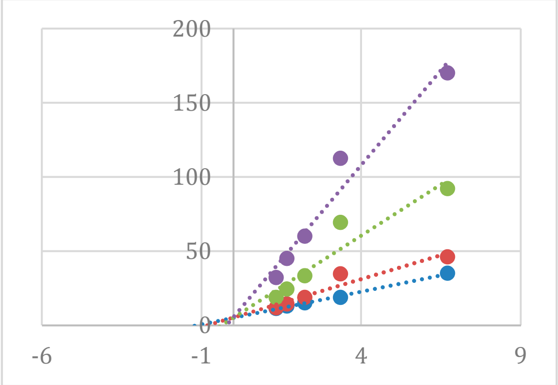
200
150
100
50
0
-6
-1
4
9

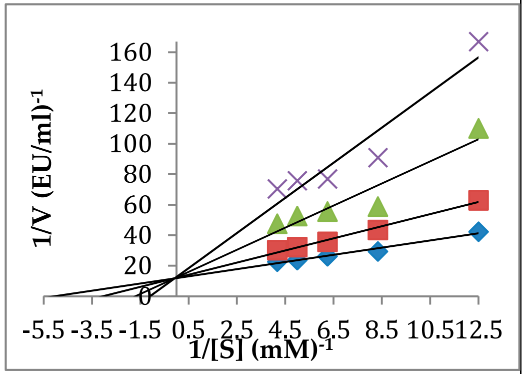
160
140
120
100
80
60
40
20
0
-5.5 -3.5 -1.5 0.5 2.5 4.5 6.5 8.5 10.5 12.5
1/V (EU/ml)-1
1/[S] (mM)-1

2k

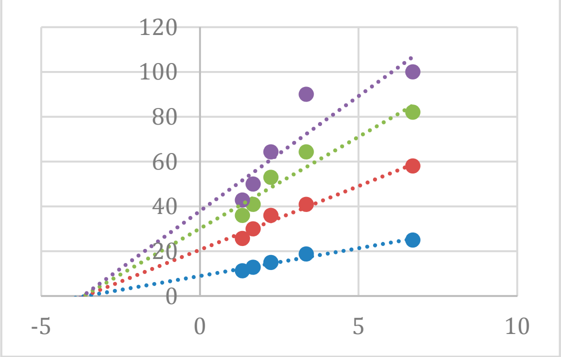
120
100
80
60
40
20
0
-5
0
5
10

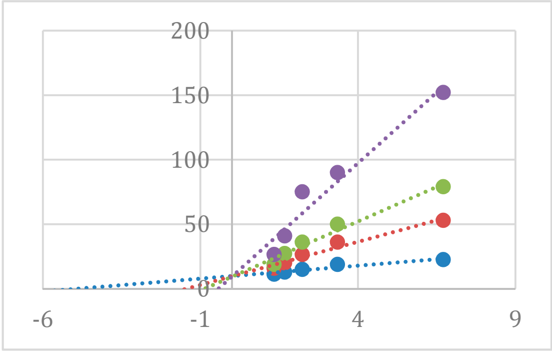
200
150
100
50
0
-6
-1
4
9

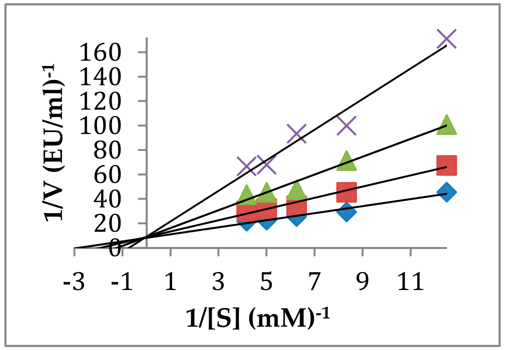
160
140
120
100
80
60
40
20
0
-3 -1 1 3 5 7 9 11
1/V (EU/ml)-1
1/[S] (mM)-1

**AZA**	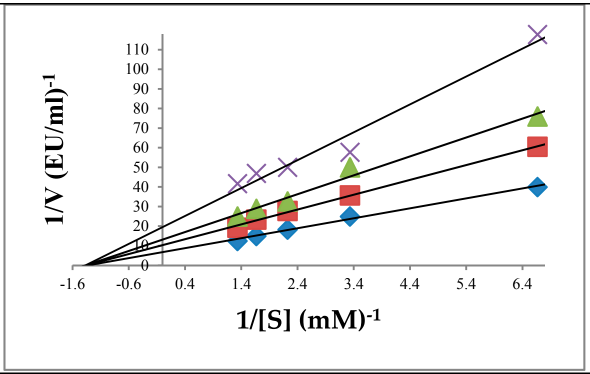	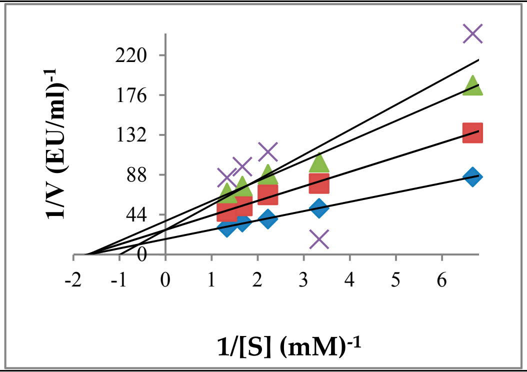	-
**TAC**	-	-	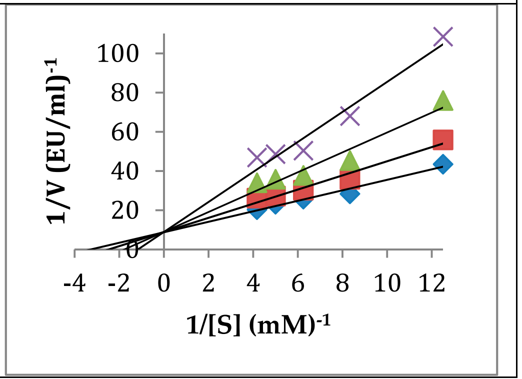
